# 2-(2,4-Dichloro­phen­yl)-2-oxoethyl 4-meth­oxy­benzoate

**DOI:** 10.1107/S1600536811048720

**Published:** 2011-11-19

**Authors:** Hoong-Kun Fun, Tze Shyang Chia, Seema Shenvi, Arun M. Isloor, B. Garudachari

**Affiliations:** a X-ray Crystallography Unit, School of Physics, Universiti Sains Malaysia, 11800 USM, Penang, Malaysia; b Medicinal Chemstry Division, Department of Chemistry, National Institute of Technology-Karnataka, Surathkal, Mangalore 575 025, India

## Abstract

In the title compound, C_16_H_12_Cl_2_O_4_, the dihedral angle between the benzene rings is 70.11 (6)°. In the crystal, mol­ecules are linked by C—H⋯O hydrogen bonds into a three-dimensional network. A C—H⋯π inter­action is also observed.

## Related literature

For related structures and background to phenacyl benzoates, see: Fun *et al.* (2011*a* ▶,*b* ▶). For reference bond lengths, see: Allen *et al.* (1987 ▶). For stability of the temperature controller used in the data collection, see: Cosier & Glazer (1986 ▶).
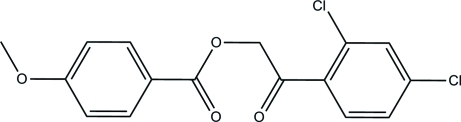


## Experimental

### 

#### Crystal data

C_16_H_12_Cl_2_O_4_
*M* *_r_* = 339.16
Monoclinic, 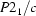

*a* = 9.0508 (1) Å
*b* = 7.0846 (1) Å
*c* = 23.3337 (3) Å
β = 102.509 (1)°
*V* = 1460.67 (3) Å^3^
*Z* = 4
Mo *K*α radiation
μ = 0.46 mm^−1^
*T* = 100 K
0.36 × 0.30 × 0.13 mm

#### Data collection

Bruker SMART APEXII CCD diffractometer
Absorption correction: multi-scan (*SADABS*; Bruker, 2009 ▶) *T*_min_ = 0.852, *T*_max_ = 0.942
24788 measured reflections
6502 independent reflections
5027 reflections with *I* > 2σ(*I*)
*R*_int_ = 0.037

#### Refinement

*R*[*F* ^2^ > 2σ(*F* ^2^)] = 0.039
*wR*(*F* ^2^) = 0.097
*S* = 1.02
6502 reflections
200 parameters
H-atom parameters constrained
Δρ_max_ = 0.52 e Å^−3^
Δρ_min_ = −0.34 e Å^−3^

### 

Data collection: *APEX2* (Bruker, 2009 ▶); cell refinement: *SAINT* (Bruker, 2009 ▶); data reduction: *SAINT*; program(s) used to solve structure: *SHELXTL* (Sheldrick, 2008 ▶); program(s) used to refine structure: *SHELXTL*; molecular graphics: *SHELXTL*; software used to prepare material for publication: *SHELXTL* and *PLATON* (Spek, 2009 ▶).

## Supplementary Material

Crystal structure: contains datablock(s) global, I. DOI: 10.1107/S1600536811048720/hb6515sup1.cif

Structure factors: contains datablock(s) I. DOI: 10.1107/S1600536811048720/hb6515Isup2.hkl

Supplementary material file. DOI: 10.1107/S1600536811048720/hb6515Isup3.cml

Additional supplementary materials:  crystallographic information; 3D view; checkCIF report

## Figures and Tables

**Table 1:** Hydrogen-bond geometry (Å, °) *Cg*1 is the centroid of the C1–C6 benzene ring.

*D*—H⋯*A*	*D*—H	H⋯*A*	*D*⋯*A*	*D*—H⋯*A*
C2—H2*A*⋯O4^i^	0.95	2.54	3.4809 (13)	173
C5—H5*A*⋯O2^ii^	0.95	2.35	3.2745 (15)	164
C8—H8*B*⋯O3^iii^	0.99	2.50	3.4124 (17)	153
C16—H16*C*⋯*Cg*1^iv^	0.98	2.84	3.5655 (15)	132

Symmetry codes: (i) 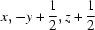; (ii) 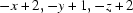; (iii) 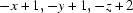; (iv) 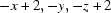.

**Fig. 1.**
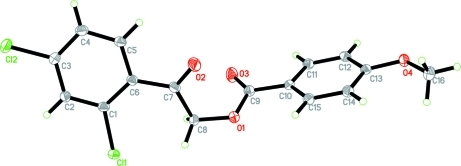
The molecular structure of the title compound with 50% probability displacement ellipsoids.

**Fig. 2.**
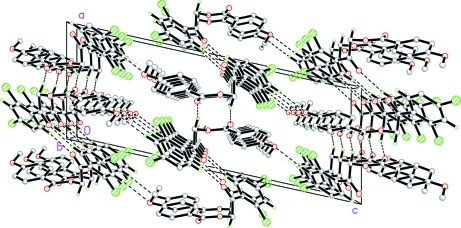
The crystal packing of the title compound. The dashed lines represent the hydrogen bonds.

## supplementary crystallographic information

### Comment

As part of our ongoing studies of phenacyl benzoates (Fun *et al.*, 2011*a*,*b*), we hereby report the crystal structure of the title compound, (I).

The molecular structure of the title compound is shown in Fig. 1. The C1–C6 benzene ring [maximum deviation of 0.008 (1) Å at atom C4] and C10–C15 benzene ring [maximum deviation of 0.005 (1) Å at atoms C12 and C15] make a dihedral angle of 70.11 (6)° with each other. Bond lengths (Allen *et al.*, 1987) and angles are within normal ranges and are comparable to related structures (Fun *et al.*, 2011*a*,*b*).

In the crystal structure (Fig. 2), the molecules are interconnected by C2—H2A···O4, C5—H5A···O2 and C8—H8B···O3 hydrogen bonds (Table 1) forming a three-dimensional network. The crystal structure is further stabilized by C—H···π interactions, involving the centroids of C1–C6 benzene rings.

### Experimental

The mixture of 4-methoxybenzoic acid (340.4 mg, 0.0022 mol), potassium carbonate (340.3 mg, 0.0025 mol) and 2-chloro-1-(2,4-dichlorophenyl) ethanone (500 mg, 0.0022 mol) in DMF (10 ml) was stirred at room temperature for 2 h. On cooling, colorless needle-shaped crystals of 2-(2,4-dichlorophenyl)-2-oxoethyl 4-methoxybenzoate begins to separate out. It was collected by filtration and then recrystallized from ethanol to yield colourless plates of (I). Yield: 691.6 mg, 92.7%, *M.p.*: 385–386 K.

### Refinement

All H atoms were positioned geometrically [C—H = 0.95, 0.98 or 0.99 Å] and refined using a riding model with *U*_iso_(H) = 1.2 or 1.5*U*_eq_(C). A rotating group model was applied to the methyl group. In the final refinement, an outlier (-3 10 5) was omitted.

### Crystal data

**Table:**

C_16_H_12_Cl_2_O_4_	*F*(000) = 696
*M**_r_* = 339.16	*D*_x_ = 1.542 Mg m^−^^3^
Monoclinic, *P*2_1_/*c*	Mo *K*α radiation, λ = 0.71073 Å
Hall symbol: -P 2ybc	Cell parameters from 6774 reflections
*a* = 9.0508 (1) Å	θ = 2.3–35.0°
*b* = 7.0846 (1) Å	µ = 0.46 mm^−^^1^
*c* = 23.3337 (3) Å	*T* = 100 K
β = 102.509 (1)°	Plate, colourless
*V* = 1460.67 (3) Å^3^	0.36 × 0.30 × 0.13 mm
*Z* = 4	

### Data collection

**Table:**

Bruker SMART APEXII CCD diffractometer	6502 independent reflections
Radiation source: fine-focus sealed tube	5027 reflections with *I* > 2σ(*I*)
graphite	*R*_int_ = 0.037
φ and ω scans	θ_max_ = 35.2°, θ_min_ = 2.3°
Absorption correction: multi-scan (*SADABS*; Bruker, 2009)	*h* = −14→12
*T*_min_ = 0.852, *T*_max_ = 0.942	*k* = −11→10
24788 measured reflections	*l* = −36→37

### Refinement

**Table:**

Refinement on *F*^2^	Primary atom site location: structure-invariant direct methods
Least-squares matrix: full	Secondary atom site location: difference Fourier map
*R*[*F*^2^ > 2σ(*F*^2^)] = 0.039	Hydrogen site location: inferred from neighbouring sites
*wR*(*F*^2^) = 0.097	H-atom parameters constrained
*S* = 1.02	*w* = 1/[σ^2^(*F*_o_^2^) + (0.040*P*)^2^ + 0.5816*P*] where *P* = (*F*_o_^2^ + 2*F*_c_^2^)/3
6502 reflections	(Δ/σ)_max_ = 0.004
200 parameters	Δρ_max_ = 0.52 e Å^−^^3^
0 restraints	Δρ_min_ = −0.34 e Å^−^^3^

### Special details

**Table:**

Experimental. The crystal was placed in the cold stream of an Oxford Cryosystems Cobra open-flow nitrogen cryostat (Cosier & Glazer, 1986) operating at 100.0 (1) K.
Geometry. All e.s.d.'s (except the e.s.d. in the dihedral angle between two l.s. planes) are estimated using the full covariance matrix. The cell e.s.d.'s are taken into account individually in the estimation of e.s.d.'s in distances, angles and torsion angles; correlations between e.s.d.'s in cell parameters are only used when they are defined by crystal symmetry. An approximate (isotropic) treatment of cell e.s.d.'s is used for estimating e.s.d.'s involving l.s. planes.
Refinement. Refinement of *F*^2^ against ALL reflections. The weighted *R*-factor *wR* and goodness of fit *S* are based on *F*^2^, conventional *R*-factors *R* are based on *F*, with *F* set to zero for negative *F*^2^. The threshold expression of *F*^2^ > σ(*F*^2^) is used only for calculating *R*-factors(gt) *etc*. and is not relevant to the choice of reflections for refinement. *R*-factors based on *F*^2^ are statistically about twice as large as those based on *F*, and *R*- factors based on ALL data will be even larger.

### Fractional atomic coordinates and isotropic or equivalent isotropic displacement parameters (Å^2^)

**Table:**

	*x*	*y*	*z*	*U*_iso_*/*U*_eq_	
Cl1	0.66881 (3)	0.47740 (4)	1.178052 (11)	0.01926 (7)	
Cl2	0.97532 (4)	1.12299 (5)	1.190485 (14)	0.02429 (7)	
O1	0.65268 (10)	0.12541 (13)	1.01931 (3)	0.01802 (16)	
O2	0.89431 (11)	0.33741 (15)	1.03290 (4)	0.0262 (2)	
O3	0.56227 (12)	0.33618 (14)	0.94784 (4)	0.0265 (2)	
O4	0.67141 (9)	−0.37683 (13)	0.79718 (3)	0.01786 (16)	
C1	0.78521 (12)	0.62113 (17)	1.14699 (5)	0.01504 (19)	
C2	0.82728 (12)	0.79099 (17)	1.17576 (5)	0.01596 (19)	
H2A	0.7914	0.8237	1.2098	0.019*	
C3	0.92243 (12)	0.91200 (17)	1.15397 (5)	0.0168 (2)	
C4	0.97624 (13)	0.86750 (18)	1.10412 (5)	0.0186 (2)	
H4A	1.0399	0.9525	1.0892	0.022*	
C5	0.93461 (12)	0.69604 (18)	1.07679 (5)	0.0166 (2)	
H5A	0.9726	0.6634	1.0432	0.020*	
C6	0.83829 (12)	0.56876 (17)	1.09690 (4)	0.01481 (19)	
C7	0.80621 (12)	0.38880 (18)	1.06248 (5)	0.0167 (2)	
C8	0.66353 (13)	0.27437 (18)	1.06150 (5)	0.0177 (2)	
H8A	0.6667	0.2205	1.1009	0.021*	
H8B	0.5736	0.3572	1.0511	0.021*	
C9	0.60826 (13)	0.17893 (18)	0.96250 (5)	0.0173 (2)	
C10	0.62482 (12)	0.02595 (17)	0.92132 (4)	0.01478 (18)	
C11	0.56119 (12)	0.05046 (17)	0.86144 (5)	0.01633 (19)	
H11A	0.5058	0.1620	0.8484	0.020*	
C12	0.57849 (12)	−0.08669 (18)	0.82128 (5)	0.0163 (2)	
H12A	0.5342	−0.0699	0.7808	0.020*	
C13	0.66111 (12)	−0.24992 (17)	0.84031 (5)	0.01497 (19)	
C14	0.72520 (13)	−0.27659 (18)	0.89986 (5)	0.0168 (2)	
H14A	0.7814	−0.3876	0.9129	0.020*	
C15	0.70546 (13)	−0.13853 (17)	0.93974 (5)	0.0166 (2)	
H15A	0.7477	−0.1566	0.9803	0.020*	
C16	0.75581 (14)	−0.54602 (19)	0.81472 (5)	0.0216 (2)	
H16A	0.7483	−0.6293	0.7807	0.032*	
H16B	0.7147	−0.6104	0.8450	0.032*	
H16C	0.8622	−0.5140	0.8305	0.032*	

### Atomic displacement parameters (Å^2^)

**Table:**

	*U*^11^	*U*^22^	*U*^33^	*U*^12^	*U*^13^	*U*^23^
Cl1	0.02234 (12)	0.01956 (14)	0.01897 (11)	−0.00520 (10)	0.01125 (9)	−0.00190 (10)
Cl2	0.02641 (14)	0.01913 (15)	0.02799 (14)	−0.00677 (11)	0.00735 (11)	−0.00626 (11)
O1	0.0223 (4)	0.0167 (4)	0.0153 (3)	−0.0022 (3)	0.0047 (3)	−0.0019 (3)
O2	0.0247 (4)	0.0287 (5)	0.0300 (4)	−0.0050 (4)	0.0162 (4)	−0.0101 (4)
O3	0.0382 (5)	0.0181 (5)	0.0227 (4)	0.0068 (4)	0.0051 (4)	−0.0010 (3)
O4	0.0203 (4)	0.0166 (4)	0.0168 (3)	0.0010 (3)	0.0041 (3)	−0.0025 (3)
C1	0.0136 (4)	0.0171 (5)	0.0151 (4)	−0.0018 (4)	0.0047 (3)	0.0008 (4)
C2	0.0160 (4)	0.0166 (5)	0.0154 (4)	−0.0001 (4)	0.0036 (3)	−0.0011 (4)
C3	0.0161 (4)	0.0151 (5)	0.0186 (4)	−0.0009 (4)	0.0026 (3)	−0.0003 (4)
C4	0.0182 (5)	0.0189 (6)	0.0194 (5)	−0.0026 (4)	0.0061 (4)	0.0017 (4)
C5	0.0156 (4)	0.0195 (6)	0.0157 (4)	−0.0005 (4)	0.0056 (3)	0.0011 (4)
C6	0.0138 (4)	0.0170 (5)	0.0139 (4)	−0.0008 (4)	0.0035 (3)	−0.0004 (4)
C7	0.0166 (4)	0.0190 (6)	0.0152 (4)	−0.0012 (4)	0.0051 (3)	−0.0008 (4)
C8	0.0196 (5)	0.0177 (6)	0.0170 (4)	−0.0037 (4)	0.0069 (4)	−0.0040 (4)
C9	0.0174 (5)	0.0179 (5)	0.0168 (4)	−0.0015 (4)	0.0044 (4)	−0.0012 (4)
C10	0.0152 (4)	0.0143 (5)	0.0151 (4)	−0.0022 (4)	0.0039 (3)	−0.0001 (4)
C11	0.0165 (4)	0.0161 (5)	0.0165 (4)	0.0008 (4)	0.0037 (3)	0.0018 (4)
C12	0.0163 (4)	0.0186 (6)	0.0139 (4)	0.0002 (4)	0.0028 (3)	0.0015 (4)
C13	0.0140 (4)	0.0158 (5)	0.0156 (4)	−0.0024 (4)	0.0041 (3)	−0.0001 (4)
C14	0.0181 (5)	0.0154 (5)	0.0164 (4)	0.0002 (4)	0.0027 (3)	0.0007 (4)
C15	0.0179 (5)	0.0174 (5)	0.0139 (4)	−0.0006 (4)	0.0021 (3)	0.0007 (4)
C16	0.0243 (5)	0.0167 (6)	0.0242 (5)	0.0015 (4)	0.0061 (4)	−0.0013 (4)

### Geometric parameters (Å, °)

**Table:**

Cl1—C1	1.7329 (11)	C7—C8	1.5207 (16)
Cl2—C3	1.7357 (13)	C8—H8A	0.9900
O1—C9	1.3535 (14)	C8—H8B	0.9900
O1—C8	1.4320 (14)	C9—C10	1.4774 (16)
O2—C7	1.2178 (13)	C10—C15	1.3927 (16)
O3—C9	1.2123 (15)	C10—C11	1.4019 (15)
O4—C13	1.3677 (14)	C11—C12	1.3821 (16)
O4—C16	1.4326 (16)	C11—H11A	0.9500
C1—C2	1.3904 (16)	C12—C13	1.3963 (17)
C1—C6	1.4063 (14)	C12—H12A	0.9500
C2—C3	1.3874 (16)	C13—C14	1.3980 (15)
C2—H2A	0.9500	C14—C15	1.3878 (16)
C3—C4	1.3912 (16)	C14—H14A	0.9500
C4—C5	1.3857 (17)	C15—H15A	0.9500
C4—H4A	0.9500	C16—H16A	0.9800
C5—C6	1.4037 (16)	C16—H16B	0.9800
C5—H5A	0.9500	C16—H16C	0.9800
C6—C7	1.5013 (17)		
			
C9—O1—C8	115.37 (10)	H8A—C8—H8B	108.2
C13—O4—C16	117.18 (9)	O3—C9—O1	123.00 (11)
C2—C1—C6	121.51 (10)	O3—C9—C10	124.59 (10)
C2—C1—Cl1	115.74 (8)	O1—C9—C10	112.40 (10)
C6—C1—Cl1	122.73 (9)	C15—C10—C11	119.07 (10)
C3—C2—C1	118.99 (10)	C15—C10—C9	122.23 (10)
C3—C2—H2A	120.5	C11—C10—C9	118.67 (10)
C1—C2—H2A	120.5	C12—C11—C10	120.42 (11)
C2—C3—C4	121.60 (11)	C12—C11—H11A	119.8
C2—C3—Cl2	118.64 (9)	C10—C11—H11A	119.8
C4—C3—Cl2	119.76 (9)	C11—C12—C13	119.88 (10)
C5—C4—C3	118.29 (11)	C11—C12—H12A	120.1
C5—C4—H4A	120.9	C13—C12—H12A	120.1
C3—C4—H4A	120.9	O4—C13—C12	115.32 (9)
C4—C5—C6	122.41 (10)	O4—C13—C14	124.26 (11)
C4—C5—H5A	118.8	C12—C13—C14	120.42 (10)
C6—C5—H5A	118.8	C15—C14—C13	119.03 (11)
C5—C6—C1	117.19 (11)	C15—C14—H14A	120.5
C5—C6—C7	115.39 (9)	C13—C14—H14A	120.5
C1—C6—C7	127.40 (10)	C14—C15—C10	121.18 (10)
O2—C7—C6	118.85 (10)	C14—C15—H15A	119.4
O2—C7—C8	119.36 (11)	C10—C15—H15A	119.4
C6—C7—C8	121.73 (9)	O4—C16—H16A	109.5
O1—C8—C7	109.56 (9)	O4—C16—H16B	109.5
O1—C8—H8A	109.8	H16A—C16—H16B	109.5
C7—C8—H8A	109.8	O4—C16—H16C	109.5
O1—C8—H8B	109.8	H16A—C16—H16C	109.5
C7—C8—H8B	109.8	H16B—C16—H16C	109.5
			
C6—C1—C2—C3	0.75 (17)	C6—C7—C8—O1	172.24 (10)
Cl1—C1—C2—C3	178.95 (9)	C8—O1—C9—O3	−8.97 (16)
C1—C2—C3—C4	0.12 (17)	C8—O1—C9—C10	170.10 (9)
C1—C2—C3—Cl2	−179.57 (9)	O3—C9—C10—C15	166.89 (12)
C2—C3—C4—C5	−1.14 (17)	O1—C9—C10—C15	−12.17 (15)
Cl2—C3—C4—C5	178.55 (9)	O3—C9—C10—C11	−10.99 (17)
C3—C4—C5—C6	1.34 (17)	O1—C9—C10—C11	169.95 (10)
C4—C5—C6—C1	−0.51 (17)	C15—C10—C11—C12	−0.08 (16)
C4—C5—C6—C7	−179.40 (11)	C9—C10—C11—C12	177.86 (10)
C2—C1—C6—C5	−0.56 (16)	C10—C11—C12—C13	−0.65 (17)
Cl1—C1—C6—C5	−178.63 (8)	C16—O4—C13—C12	179.72 (10)
C2—C1—C6—C7	178.18 (11)	C16—O4—C13—C14	−1.06 (16)
Cl1—C1—C6—C7	0.11 (17)	C11—C12—C13—O4	179.94 (10)
C5—C6—C7—O2	21.81 (16)	C11—C12—C13—C14	0.69 (17)
C1—C6—C7—O2	−156.95 (12)	O4—C13—C14—C15	−179.17 (10)
C5—C6—C7—C8	−155.42 (11)	C12—C13—C14—C15	0.00 (16)
C1—C6—C7—C8	25.83 (17)	C13—C14—C15—C10	−0.75 (17)
C9—O1—C8—C7	−75.93 (12)	C11—C10—C15—C14	0.79 (17)
O2—C7—C8—O1	−4.98 (16)	C9—C10—C15—C14	−177.08 (11)

### Hydrogen-bond geometry (Å, °)

**Table:**

Cg1 is the centroid of the C1–C6 benzene ring.

**Table:**

*D*—H···*A*	*D*—H	H···*A*	*D*···*A*	*D*—H···*A*
C2—H2A···O4^i^	0.95	2.54	3.4809 (13)	173
C5—H5A···O2^ii^	0.95	2.35	3.2745 (15)	164
C8—H8B···O3^iii^	0.99	2.50	3.4124 (17)	153
C16—H16C···Cg1^iv^	0.98	2.84	3.5655 (15)	132

Symmetry codes: (i) *x*, −*y*+1/2, *z*+1/2; (ii) −*x*+2, −*y*+1, −*z*+2; (iii) −*x*+1, −*y*+1, −*z*+2; (iv) −*x*+2, −*y*, −*z*+2.
